# Association and interactions between mixed exposure to trace elements and the prevalence of kidney stones: a study of NHANES 2017–2018

**DOI:** 10.3389/fpubh.2023.1251637

**Published:** 2023-10-26

**Authors:** Xiao Wang, Jia Zhang, Zhibin Ma, Yaya Yang, Ying Dang, Shuting Cao, Xiaoru Shi, Changping Ouyang, Jinhua Pan, Xiaobin Hu

**Affiliations:** Institute of Epidemiology and Health Statistics, School of Public Health, Lanzhou University, Lanzhou, China

**Keywords:** trace element, kidney stone, NHANES, BKMR, qgcomp

## Abstract

**Background:**

The association between exposure to trace elements mixture and the prevalence of kidney stones and the interactions between elements are unclear. The aim of this study was to explore the association between exposure to trace elements mixture and the prevalence of kidney stones and the interactions between the elements.

**Methods:**

A total of 1,244 participants (139 kidney stone formers and 1,105 non-stone former participants) in NHANES 2017–2018 were included. The exposure to trace elements was evaluated by measuring their concentration in urine samples. Three methods, Logistic regression, quantile-based g computation (qgcomp), and Bayesian kernel machine regression (BKMR), were used for analysis.

**Results:**

According to the results from qgcomp and BKMR, a negative association was found between exposure to the 13 trace elements and the prevalence of kidney stones [OR = 0.50 (0.32, 0.78)]. Subgroup analysis revealed that Co, As, and iodine in the whole population, Co, As, and Ni in males, and Cs, iodine, and Sb in females, were most strongly associated with kidney stones. Kidney stone was found to be positively correlated with Co and negatively correlated with the other elements. Besides, there were significant interactions between Ni and Pb in the whole population, Co and iodine in males, and Pb and iodine in females.

**Conclusion:**

There was a negative association between exposure to the mixture of 13 trace elements and the prevalence of kidney stones.

## 1. Introduction

Kidney stones are widely prevalent, and the prevalence of kidney stones has shown an increasing trend over the years worldwide. The self-reported prevalence of kidney stones among adults aged 20 years and older in the United States was 3.8, 5.2% (1), 8.8% (2), and 11.0% (3) in 1976–1980, 1988–1994, 2007–2010, and 2015–2018, respectively. From 1976 to 2018, the prevalence increased nearly threefold. The incidence of kidney stones ranges from 114 to 720 per 100,000 persons, with a prevalence of 1.7 to 14.8%, and the incidence is rising in almost all nations, according to a retrospective study of epidemiologic data from seven countries (4). While kidney stones do not directly raise mortality rates in the general population (5), they are linked with excruciating pain, are prone to recurrence (6), and increase the risk of myocardial infarction in people who have them (7). In addition, in overweight people, kidney stones may also lead to renal impairment (8). All of the evidence above suggests that kidney stones need to be taken seriously.

Many factors contribute to the development of kidney stones, among which nutritional factors are very important (9). Reduced dietary calcium (Ca) intake (10, 11) and decreased urine output caused by insufficient water intake (12) are significant risk factors for kidney stone development, according to prior research. In addition to macronutrients such as calcium, which have an impact on the risk of kidney stones, exposure to many trace elements may also have an impact on kidney stones, for example, elevated dietary zinc (Zn) intake may be a protective factor for the risk of kidney stones (13, 14), and there may be an association between exposure concentrations of heavy metals such as cadmium (Cd), lead (Pb), arsenic (As), mercury (Hg), trimethyltin and the risk of kidney stones (15–18). The precise relationship between kidney stones and trace elements is unclear, due to discrepancies between recent studies on the topic. Human exposure to numerous trace elements is diverse and complicated, but prior research has only looked at a few hazardous trace elements, ignoring other less toxic or beneficial elements or mixed exposures to several trace elements, which may not accurately reflect actual exposure.

This study was based on data from the National Health and Nutrition Examination Survey (NHANES) 2017–2018. The impact of single trace elements was analyzed using multivariable logistic regression, and quantile-based g computation (qgcomp) and Bayesian kernel machine regression (BKMR) were applied to investigate the roles of trace elements co-exposure in kidney stones and the interactions between the trace elements.

## 2. Materials and methods

### 2.1. Study population

All study subjects in this study were from NHANES, an ongoing cross-sectional survey of nationally representative samples of the US civilian non-institutionalized population while using a complex multi-stage sampling method. The study analyzed data from the 2017–2018 NHANES cycle because urine nickel (Ni) was detected only in this cycle.

**Figure 1:**
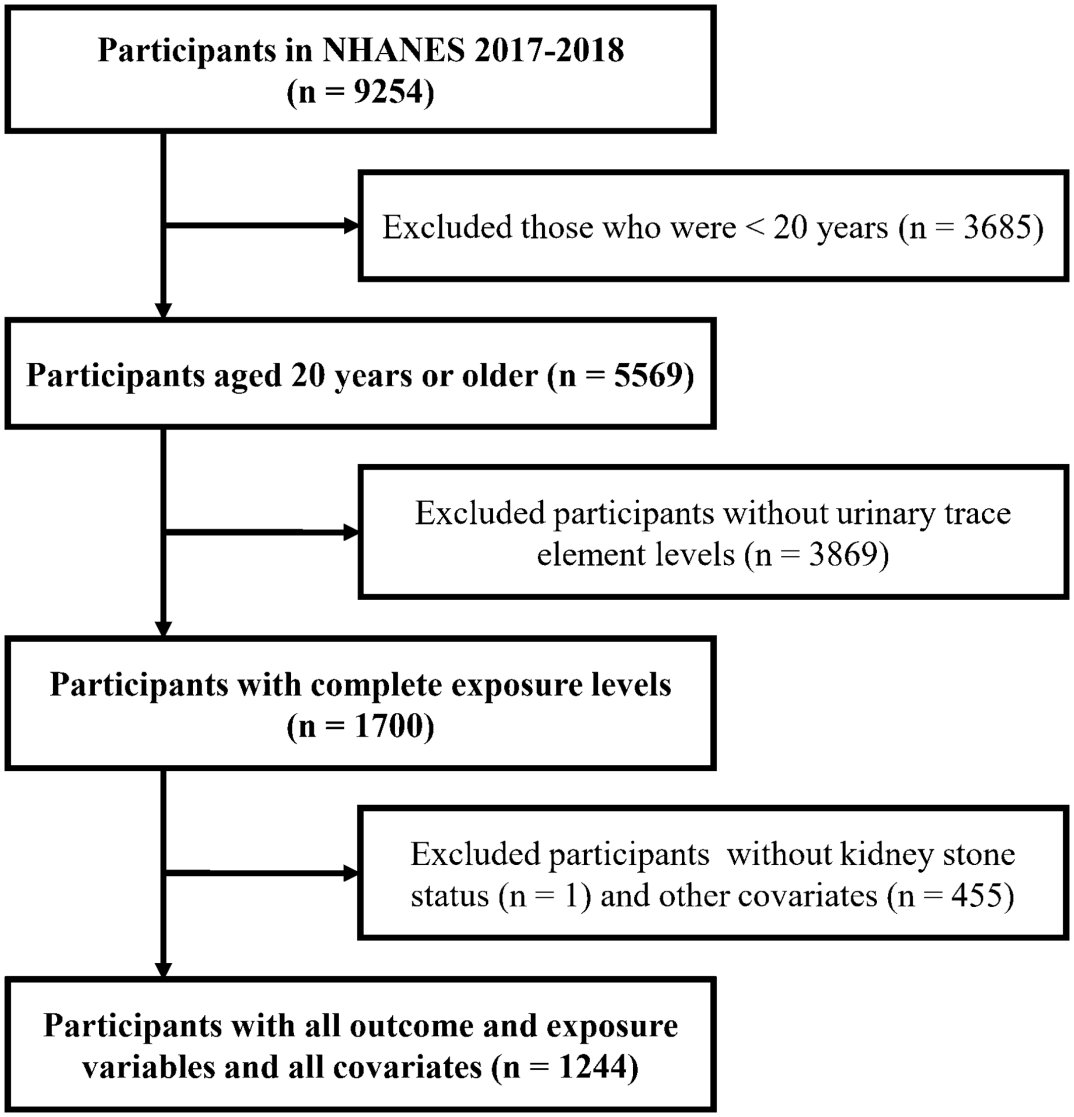
Flowchart of the selection process for eligible participants.

This cross-sectional study was performed under the Strengthening the Reporting of Observational Studies in Epidemiology (STROBE) guidelines (Supplementary Table S1). Participants were eligible if they met the following inclusion criteria: (1) aged above 20 years and (2) had available kidney stone status, urinary trace element measurements and all covariates. Figure 1 depicts a detailed flowchart of the selection process for eligible participants. Ultimately, 1,244 participants were enrolled in the study.

### 2.2. Assessment of kidney stones

Information on kidney stones was collected by the NHANES questionnaire. All adults older than 20 years were asked “Have you ever had kidney stones?” Subjects who answered “yes” were considered to be kidney stone patients.

### 2.3. Trace element measurement

The detection results of a total of 13 trace elements in urine were obtained from the 2017–2018 cycle of the NHANES. The urinary trace elements include Ni, molybdenum (Mo), thallium (Tl), Pb, barium (Ba), cobalt (Co), cesium (Cs), antimony (Sb), tin (Sn), tungsten (Tu), As, iodine (I), and Cd. Iodine was measured by inductively coupled plasma dynamic reaction cell mass spectrometry (ICP-DRC-MS) and the remaining elements were measured by inductively coupled plasma mass spectrometry (ICP-MS). For concentrations below the limit of detection (LOD), we used an imputed value of LOD/ √2 according to the NHANES standard. The concentrations of metals in urine were all corrected by urinary creatinine in our analysis and were expressed as μg/g creatinine.

### 2.4. Covariates

According to previous studies, the following variables were included as covariates (19, 20): age, gender (male or female), race/ethnicity (Mexican American, Other Hispanic, non-Hispanic white, non-Hispanic black, other race/multiracial), education (under high school, high school or equivalent, above high school), the ratio of family income to poverty (PIR), marital status (married/cohabiting, widowed/divorced/separated, never married), drinking alcohol status (never, ever), body mass index (BMI), serum cotinine, and intakes of total energy, calcium, phosphate, sodium, potassium, magnesium, total fluid, alcohol, caffeine, and vitamins B6, C, and D. All covariates were collected by questionnaires (both demographic and dietary questionnaires) except for serum cotinine, which was measured by an isotope-dilution high-performance liquid chromatography-mass spectrometric (ID HPLC-MS) method. The average intakes of the above dietary factors from the two 24-h recalls were used in this analysis, or only the first day’s 24-h recall was applied if the second day’s recall was not available.

### 2.5. Statistical analysis

The statistical analysis accounted for the complex sample design of NHANES (21), using SPSS (version 26) and R (version 4.2.1), and Subsample A weight (WTSA2YR), which was the measurement weight of the urinary metal, was used as sample weight (22). For continuous variables, the Shapiro–Wilk test was used to explore whether the variables conformed to a normal distribution. Variables that conformed to a normal distribution were described by the mean ± SD, and those that did not conform to a normal distribution were described by the median [P25, P75]. Categorical variables were presented as No. (%). The urinary trace element concentrations were natural logarithm (Ln)-transformed, and Spearman correlation analysis was used to examine the correlations between each urinary trace element concentration. Subgroup analysis was stratified by gender.

Urinary trace element concentrations were divided into four quartiles (Q1, Q2, Q3, and Q4). The relationships between single elements and kidney stones were estimated by survey-weighted multivariable logistic regression, shown as odds ratios (OR) and 95% confidence intervals (CI) [OR, (95%CI)]. A linear trend test was performed with the median value of each quartile treated as a continuous variable in the models.

Qgcomp can be used to assess the overall effect of multiple exposures on outcomes and to identify primary exposures (23). Qgcomp indices (ranging from-1 to 1) can be used to assess the strength and direction of the effect of trace elements mixture exposure on the odds of kidney stones. In addition, the relative weights of positive or negative associations between each trace element and kidney stones can be obtained using this method. However, the absolute values of relative weights are not directly comparable between positive and negative effects. The absolute weights of each trace element were obtained by multiplying each relative weight with the importance parameter of the positive/negative effect, and can be used to compare the importance of all elements on the effect of kidney stones. The qgcomp analysis was performed using the “qgcomp” package in R.

Given the potential non-linearity and non-additive dose–response relations among mixture exposure, BKMR was used to assess the joint effect of all trace elements on kidney stones (24). This method integrates Bayesian and statistical learning methods to estimate the nonlinear and/or interactions in the exposure-outcome association. Analyses were conducted using the bkmr package in R statistical software (25). We implemented the BKMR variable selection model with 50,000 iterations using a Markov chain Monte Carlo algorithm. We analyzed the dose–response relationships of single trace elements and the mixed exposure effects using BKMR. We examined whether there were two-way interactions among the primary exposures. We estimated an interactive effect as the difference in the first exposure health effect when the second exposure was fixed at the 90th percentile compared to fixing at the 10th percentile, and the remaining exposures were fixed to their median values. Due to the complexity of qgcomp and BKMR and the limitations of the survey package, we did not consider weights in our analysis with these two methods.

In this study, *p* < 0.05 was considered statistically significant.

Besides, multivariate imputation by chained equations (MICE) was applied to all missing covariates to test the bias of omitting observations with missing values by qgcomp.

## 3. Results

### 3.1. Basic characteristics and trace elements distribution

Among the 1,244 participants from the 2017–2018 NHANES, 139 (11.17%) were diagnosed with kidney stones. Table 1 presents the basic characteristics of adults with or without kidney stones in the United States.

**Table 1:** Basic characteristics of participants by kidney stone in the U.S.adults, NHANES 2017–2018.

Variable	Total *N* = 1,244	Kidney stone *N* = 139	Non-kidney stone *N* = 1,105
Age	52.2 ± 16.9	54.5 ± 16.0	51.9 ± 17.0
Gender			
Male	610 (49.04%)	78 (56.12%)	532 (48.14%)
Female	634 (50.96%)	61 (43.88%)	573 (51.86%)
Race/ethnicity			
Mexican American	156 (12.54%)	9 (6.47%)	147 (13.30%)
Other Hispanic	104 (8.36%)	12 (8.63%)	92 (8.33%)
Non-Hispanic White	476 (38.26%)	72 (51.80%)	404 (36.56%)
Non-Hispanic Black	282 (22.67%)	24 (17.27%)	258 (23.35%)
Other race/multiracial	226 (18.17%)	22 (15.83%)	204 (18.46%)
Education			
Under high school	218 (17.52%)	16 (11.51%)	202 (18.28%)
High school or equivalent	324 (26.05%)	36 (25.90%)	288 (26.06%)
Above high school	702 (56.43%)	87 (62.59%)	615 (55.66%)
Marital status			
Married/cohabiting	770 (61.90%)	83 (59.71%)	687 (62.17%)
Widowed/divorced/separated	304 (24.40%)	31 (22.30%)	273 (24.71%)
Never married	170 (13.67%)	25 (17.99%)	145 (13.12%)
Family PIR	2.2 [1.2, 4.1]	2.2 [1.2, 4.6]	2.2 [1.2, 4.1]
Drinking alcohol status			
Never	116 (9.32%)	12 (8.63%)	104 (9.41%)
Ever	1,128 (90.68%)	127 (91.37%)	1,001 (90.59%)
BMI (kg/m^2^)	30.1 ± 7.2	30.9 ± 7.0	30.0 ± 7.3
Serum cotinine (ng/ml)	0.03 [0.01, 4.45]	0.04 [0.01, 23.9]	0.03 [0.01, 4.17]
Intake			
Vitamin B6 (mg)	1.77 [1.23, 2.42]	1.66 [1.18, 2.32]	1.79 [1.24, 2.43]
Vitamin C (mg)	57.33 [26.56, 104.69]	47.45 [24.10, 99.00]	58.70 [27.10, 105.85]
Vitamin D (mcg)	3.30 [1.60, 5.65]	3.25 [1.55, 5.55]	3.30 [1.60, 5.68]
Ca (g)	0.83 [0.55, 1.14]	0.83 [0.58, 1.15]	0.84 [0.55, 1.14]
P (g)	1.25 [0.91, 1.66]	1.22 [0.87, 1.63]	1.25 [0.92, 1.67]
Mg (g)	0.27 [0.20, 0.37]	0.26 [0.20, 0.36]	0.27 [0.20, 0.37]
Na (g)	3.12 [2.22, 4.13]	3.29 [2.24, 4.13]	3.12 [2.22, 4.13]
K (g)	2.40 [1.79, 3.17]	2.44 [1.76, 3.09]	2.39 [1.79, 3.18]
Caffeine (mg)	98.75 [27.00, 199.00]	107.50 [29.50, 227.00]	98.50 [26.50, 196.75]
Water (L)	2.56 [1.92, 3.46]	2.58 [2.02, 3.60]	2.56 [1.90, 3.45]
Total energy (1,000 kcal)	0.20 [0.14, 0.27]	1.89 [1.36, 2.73]	1.98 [1.42, 2.66]
Urinary concentration			
Ni (ug/g creatinine)	1.13 [0.74, 1.75]	1.08 [0.70, 1.70]	1.14 [0.75, 1.76]
Mo (ug/g creatinine)	34.12 [21.94, 51.40]	31.08 [21.66, 44.02]	34.39 [22.02, 52.15]
Tl (ug/g creatinine)	0.16 [0.11, 0.22]	0.15 [0.10, 0.19]	0.16 [0.11, 0.23]
Pb (ug/g creatinine)	0.33 [0.20, 0.53]	0.33 [0.19, 0.36]	0.34 [0.20, 0.53]
Ba (ug/g creatinine)	0.99 [0.50, 1.91]	1.07 [0.62, 2.00]	0.98 [0.48, 1.90]
Co (ug/g creatinine)	0.39 [0.26, 0.58]	0.40 [0.30, 0.55]	0.38 [0.25, 0.59]
Cs (ug/g creatinine)	4.24 [3.10, 5.90]	3.88 [2.78, 4.90]	4.29 [3.14, 5.99]
Sb (ug/g creatinine)	0.04 [0.03, 0.07]	0.04 [0.03, 0.06]	0.04 [0.03, 0.07]
Sn (ug/g creatinine)	0.49 [0.26, 0.96]	0.44 [0.26, 0.83]	0.50 [0.26, 0.96]
Tu (ug/g creatinine)	0.06 [0.03, 0.09]	0.05 [0.04, 0.09]	0.06 [0.03, 0.10]
As (ug/g creatinine)	5.94 [3.39, 13.58]	4.17 [2.72, 10.74]	6.15 [3.52, 13.93]
I (g/g creatinine)	0.11 [0.07, 0.21]	0.10 [0.06, 0.17]	0.12 [0.07, 0.22]
Cd (ug/g creatinine)	0.22 [0.12, 0.42]	0.22 [0.12, 0.40]	0.23 [0.12, 0.43]

BMI, the body mass index; Family PIR, the ratio of family income to poverty.

The detection rates, median and interquartile range of each trace element are shown in Supplementary Table S2, and the detection rates of all elements were higher than 75%. Spearman correlation analysis (Supplementary Figure S1) revealed a strong correlation between Tl and Cs (*r* = 0.64). Moderate correlations were observed between Co and Ni (*r* = 0.51), Co and Ba (*r* = 0.49), and Pb and Cd (*r* = 0.43). There were only minor relationships between the other urine trace components (all *r* < 0.4).

### 3.2. Logistic regression results

Table 2 presents the survey-weighted logistic regression results. After dividing the data into four quartiles, it was found that compared with the first quartile (Q1) of each element, Mo [Q2: OR = 2.11 (1.09, 4.10)], Ba [Q2: OR = 3.19 (1.56, 6.51); Q4: OR = 2.50 (1.41, 4.44)], Co [Q2: OR = 4.22 (1.53, 11.68); Q3: OR = 3.69 (1.59, 8.58)] and Sn [Q2: OR = 1.73 (1.02, 2.95)] were positively associated with the odds of kidney stones. While Tl [Q4: OR = 0.19 (0.06, 0.59)], Cs [Q2: OR = 0.65 (0.43, 0.99); Q4: OR = 0.30 (0.15, 0.59)], As [Q2: OR = 0.45 (0.22, 0.94); Q3: OR = 0.49 (0.25, 0.98)], and iodine [Q4: OR = 0.32 (0.12, 0.85)] were negatively associated with the odds of kidney stones when these trace elements were present at certain exposure levels. Besides, the results of the trend test were significant on the association between Ba, Co, and Cs, and the odds of kidney stones (*p* < 0.05).

**Table 2:** Associations of single urinary trace elements with kidney stones and stratified by gender, NHANES, 2017–2018.

Trace element	Gender	Q1	Q2 OR (95% CI)	Q3 OR (95% CI)	Q4 OR (95% CI)	*P* for trend
Ni	Total	Ref	0.74 (0.37, 1.49)	0.57 (0.30, 1.07)	0.66 (0.39, 1.13)	0.196
	Male	Ref	**0.41 (0.23, 0.71)**	**0.33 (0.15, 0.74)**	**0.23 (0.08, 0.68)**	0.075
	Female	Ref	1.48 (0.45, 4.83)	1.24 (0.38, 4.09)	1.47 (0.50, 4.31)	0.820
Mo	Total	Ref	**2.11 (1.09, 4.10)**	1.38 (0.75, 2.54)	1.06 (0.52, 2.18)	0.344
	Male	Ref	2.02 (0.93, 4.37)	1.47 (0.51, 4.20)	1.18 (0.46, 3.02)	0.485
	Female	Ref	1.95 (0.73, 5.20)	1.38 (0.64, 2.94)	0.78 (0.29, 2.05)	0.515
Tl	Total	Ref	0.83 (0.49, 1.42)	1.00 (0.51, 1.94)	**0.19 (0.06, 0.59)**	0.140
	Male	Ref	1.04 (0.45, 2.38)	1.11 (0.38, 3.27)	0.32 (0.07, 1.40)	0.537
	Female	Ref	**0.36 (0.14, 0.91)**	0.68 (0.24, 1.93)	**0.09 (0.03, 0.25)**	**0.008**
Pb	Total	Ref	0.84 (0.32, 2.17)	1.27 (0.67, 2.41)	0.88 (0.37, 2.12)	0.602
	Male	Ref	0.63 (0.17, 2.34)	0.64 (0.18, 2.24)	0.51 (0.13, 1.96)	0.893
	Female	Ref	0.53 (0.20, 1.37)	0.97 (0.31, 3.00)	0.43 (0.10, 1.87)	0.497
Ba	Total	Ref	**3.19 (1.56, 6.51)**	1.95 (0.85, 4.50)	**2.50 (1.41, 4.44)**	**0.037**
	Male	Ref	2.09 (0.92, 4.75)	1.34 (0.46, 3.92)	2.25 (0.85, 5.93)	0.207
	Female	Ref	**7.32 (3.23, 16.60)**	**3.62 (1.45, 9.09)**	**3.46 (1.35, 8.88)**	**0.004**
Co	Total	Ref	**4.22 (1.53, 11.68)**	**3.69 (1.59, 8.58)**	2.42 (0.82, 7.16)	**0.043**
	Male	Ref	**4.39 (1.29, 15.00)**	**3.33 (1.11, 9.95)**	1.84 (0.35, 9.69)	0.053
	Female	Ref	3.74 (0.64, 21.83)	3.08 (0.95, 10.01)	2.08 (0.60, 7.19)	0.417
Cs	Total	Ref	**0.65 (0.43, 0.99)**	0.87 (0.35, 2.18)	**0.30 (0.15, 0.59)**	**0.016**
	Male	Ref	0.58 (0.30, 1.11)	0.99 (0.29, 3.40)	0.35 (0.12, 1.03)	0.263
	Female	Ref	**0.35 (0.15, 0.81)**	**0.28 (0.10, 0.77)**	**0.09 (0.04, 0.26)**	**0.013**
Sb	Total	Ref	1.83 (0.77, 4.34)	1.38 (0.71, 2.68)	0.76 (0.36, 1.62)	0.363
	Male	Ref	2.63 (0.83, 8.37)	1.86 (0.61, 5.68)	0.72 (0.23, 2.27)	0.155
	Female	Ref	1.00 (0.29, 3.42)	0.76 (0.26, 2.19)	0.61 (0.32, 1.17)	0.504
Sn	Total	Ref	**1.73 (1.02, 2.95)**	0.78 (0.42, 1.45)	1.57 (0.79, 3.11)	0.199
	Male	Ref	1.48 (0.79, 2.79)	**0.27 (0.11, 0.64)**	1.36 (0.40, 4.68)	**0.041**
	Female	Ref	1.93 (0.41, 9.05)	1.02 (0.40, 2.59)	1.00 (0.21, 4.77)	0.385
Tu	Total	Ref	1.66 (0.83, 3.31)	1.17 (0.45, 3.02)	1.32 (0.66, 2.63)	0.409
	Male	Ref	1.07 (0.42, 2.73)	0.94 (0.28, 3.12)	1.45 (0.46, 4.57)	0.920
	Female	Ref	**4.60 (1.45, 14.54)**	1.56 (0.37, 6.59)	1.68 (0.55, 5.13)	**0.023**
As	Total	Ref	**0.45 (0.22, 0.94)**	**0.49 (0.25, 0.98)**	0.61 (0.30, 1.25)	0.095
	Male	Ref	**0.21 (0.10, 0.46)**	0.43 (0.16, 1.16)	0.52 (0.18, 1.54)	**0.024**
	Female	Ref	0.87 (0.36, 2.13)	0.61 (0.25, 1.49)	0.56 (0.22, 1.41)	0.844
I	Total	Ref	0.60 (0.32, 1.14)	0.63 (0.30, 1.34)	**0.32 (0.12, 0.85)**	0.242
	Male	Ref	0.44 (0.18, 1.09)	0.38 (0.10, 1.52)	0.25 (0.06, 1.06)	0.348
	Female	Ref	0.79 (0.40, 1.54)	0.65 (0.29, 1.44)	0.28 (0.08, 1.02)	0.484
Cd	Total	Ref	1.64 (0.60, 4.53)	1.87 (0.83, 4.22)	1.87 (0.80, 4.34)	0.889
	Male	Ref	1.95 (0.51, 7.50)	1.12 (0.31, 4.08)	1.79 (0.30, 10.77)	0.756
	Female	Ref	0.31 (0.09, 1.00)	0.66 (0.30, 1.41)	**0.36 (0.16, 0.80)**	0.060

Models were adjusted for age, race/ethnicity, education, PIR, marital status, drinking alcohol status, serum cotinine, BMI, the intake of total energy, Ca, K, Na, P, Mg, water, caffeine, and vitamin B6, C and D.

Ref, reference.Bold, statistically significant (*p* < 0.05).

Subgroup analysis by gender is also displayed in Table 2. In males, after dividing trace element concentrations into quartiles, Ni [Q2: OR = 0.41 (0.23, 0.71); Q3: 0.33 (0.15, 0.74); Q4: 0.23 (0.08, 0.68)], Sn [Q3: OR = 0.27 (0.11, 0.64)], and As [Q2: OR = 0.21 (0.10, 0.46)] were found to be negatively associated with kidney stones at certain exposure levels, and Co was found to be positively associated with the odds of kidney stones [Q2: OR = 4.39 (1.29, 15.00); Q3: OR = 3.33 (1.11, 9.95)]. The results of the trend test were significant on the association between Sn, and As, and the odds of kidney stones (*p* < 0.05).

In women, it was found that Ba [Q2: OR = 7.32 (3.23, 16.60); Q3: OR = 3.62 (1.45, 9.09); Q4: OR = 3.46 (1.35, 8.88)] and Tu [Q2: OR = 4.60 (1.45, 14.54)] were risk factors for kidney stones, and Tl [Q2: OR = 0.36 (0.14, 0.91); Q4: OR = 0.09 (0.03, 0.25)], Cs [Q2: OR = 0.35 (0.15, 0.81); Q3: OR = 0.28 (0.10, 0.77); Q4: OR = 0.09 (0.04, 0.26)], and Cd [Q4: OR = 0.36 (0.16, 0.80)] were negatively associated with kidney stones when these trace elements were present at certain exposure levels. The results of the trend test were significant on the association between Tl, Ba, Cs, and Tu, and the odds of kidney stones (*p* < 0.05) (Table 2).

### 3.3. Analysis of the association of trace elements co-exposure and kidney stones by the qgcomp model

**Figure 2:**
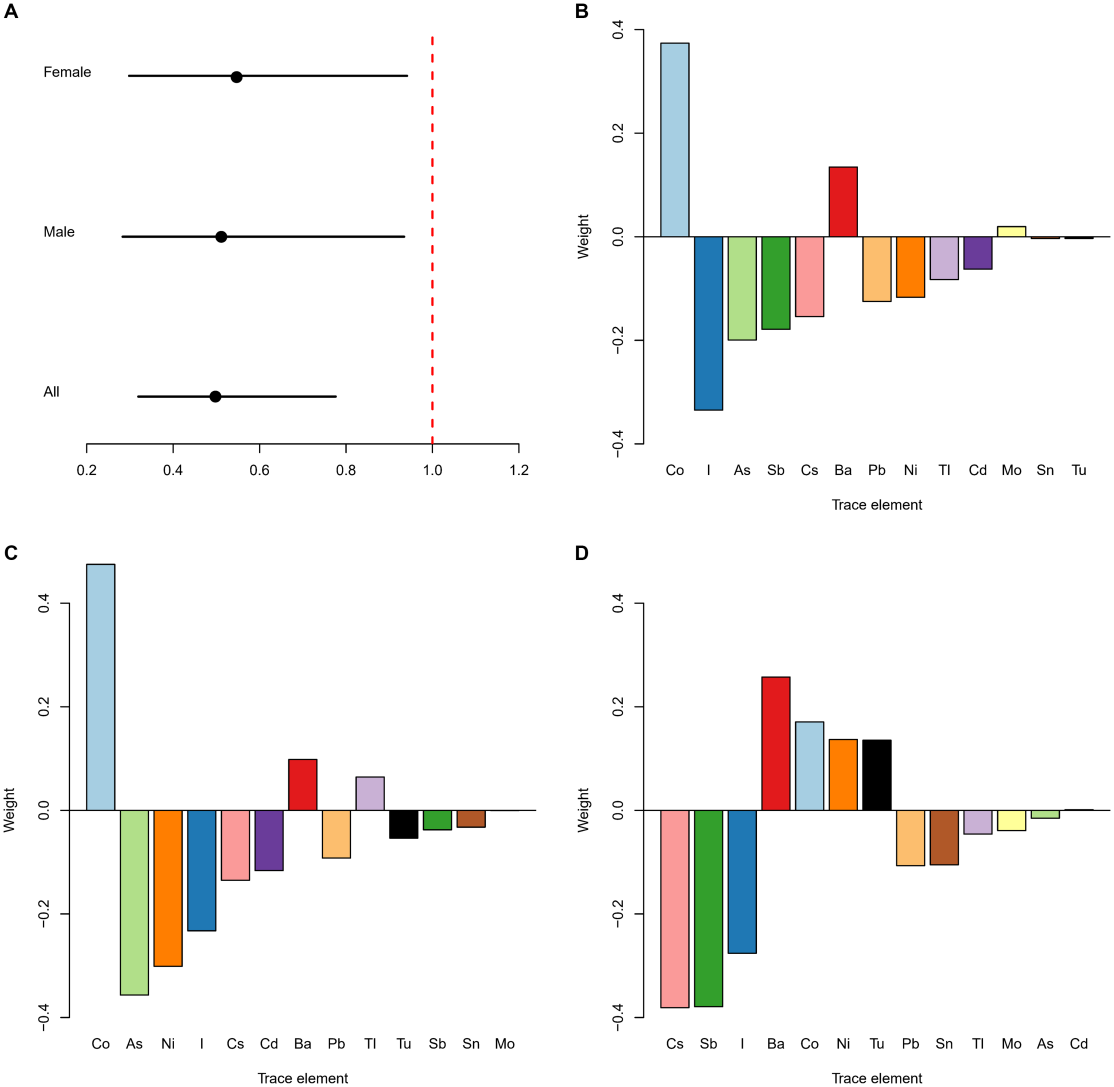
Estimated OR and weighted values of trace elements for kidney stones by qgcomp models. Associations of urinary trace elements with kidney stones odds in total population and different subgroups **(A)**. Weighted values of urinary trace elements for kidney stones in qgcomp models for total population **(B)**, males **(C)** and females **(D)**. Models (total) were adjusted for gender, age, race/ethnicity, education, PIR, marital status, drinking alcohol status, serum cotinine, BMI, the intake of total energy, Ca, K, Na, P, Mg, water, caffeine, and vitamin B6, C and D.

As shown in Figure 2A, the OR of the trace elements was negatively associated with kidney stones in all [0.50 (0.32, 0.78)], male [0.51 (0.28, 0.93)] and female participants [0.54 (0.30, 0.93)], *p* < 0.05. In the whole population, the highest weight was given to Co, which was positively associated with the odds of kidney stones, followed by iodine and As, both of which were negatively associated with the odds of kidney stones (Figure 2B). In males, the trace element most significantly associated with kidney stones was Co, with a positive association, followed by As and Ni, both of which were negatively associated with the odds of kidney stones (Figure 2C). In females, the most significantly associated trace elements with kidney stones were Cs, Sb, and iodine, which were all negatively associated with the odds of kidney stones, followed by Ba, which was positively associated with the odds of kidney stones (Figure 2D).

### 3.4. Assessment of trace elements co-exposure effects and interactions using the BKMR model

**Figure 3:**
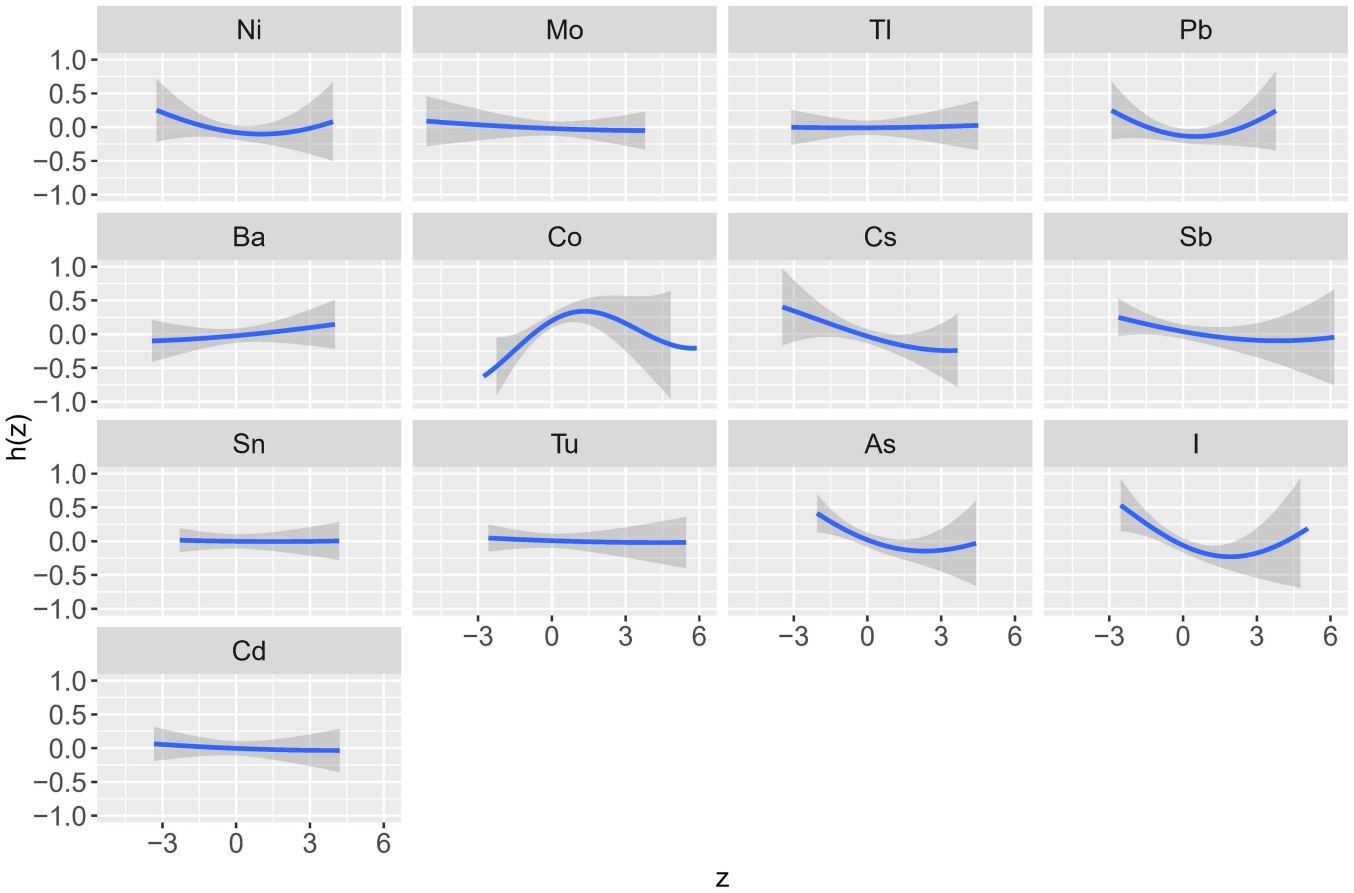
Univariate exposure-response functions for each trace element with other elements fixed at the median (total). The results were assessed by the Bayesian Kernel machine regression (BKMR) models. Models were adjusted for sex, age, race/ethnicity, education, PIR, marital status, drinking alcohol status, serum cotinine, BMI, the intake of total energy, Ca, K, Na, P, Mg, water, caffeine, and vitamin B6, C and D.

Table 3 shows the posterior inclusion probability (PIP) values for the effect of each trace element on kidney stones and Figure 3 and Supplementary Figures S2, S3 show the dose–response relationship of each trace element in the association with kidney stones. These graphs show that the trace elements with the highest PIP values (i.e., the highest contribution to kidney stones prevalence) in the overall population were Co (0.82), iodine (0.81) and As (0.66). The prevalence of kidney stones increased with rising concentrations and subsequently declined after reaching a specific threshold, forming an “inverted U-shaped” association between Co exposure and odds of kidney stones; in contrast, iodine and As exposure showed a “U-shaped” curve with the prevalence of kidney stones (Figure 3).

**Table 3:** The PIP values for each trace element by the BKMR model.

Trace element	Total PIPs	Male PIPs	Female PIPs
Ni	**0.43**	**0.47**	0.24
Mo	0.05	0.09	0.15
Tl	0.05	0.07	0.21
Pb	**0.56**	0.13	0.39
Ba	0.13	0.12	0.21
Co	**0.82**	**0.41**	0.21
Cs	**0.54**	0.12	**0.66**
Sb	0.34	0.08	**0.40**
Sn	0.05	0.10	0.11
Tu	0.06	0.21	0.11
As	**0.66**	**0.82**	0.10
I	**0.81**	0.29	**0.65**
Cd	0.07	0.20	0.13

Models were adjusted for sex, age, race/ethnicity, education, PIR, marital status, drinking alcohol status, serum cotinine, BMI, the intake of total energy, Ca, K, Na, P, Mg, water, caffeine and vitamin B6, C and D.

PIPs, the posterior inclusion probabilities.Bold, these elements had higher PIPs (≥40) and contributed more to kidney stones.

In males, the trace elements with the highest PIP values were As (0.82), Ni (0.47), and Co (0.41) (Table 3). The relationship between Co exposure and the odds of kidney stones showed an “inverted U-shaped” curve; the relationship between As exposure and the odds of kidney stones showed a “U-shaped” curve; and the relationship between Ni exposure and the odds of kidney stones showed a negative relationship (Supplementary Figure S2).

In females, the trace elements with the highest PIP values were Cs (0.66), I (0.65), and Sb (0.40), and all three exposure concentrations were negatively correlated with the odds of developing kidney stones (Supplementary Figures S3).

**Figure 4:**
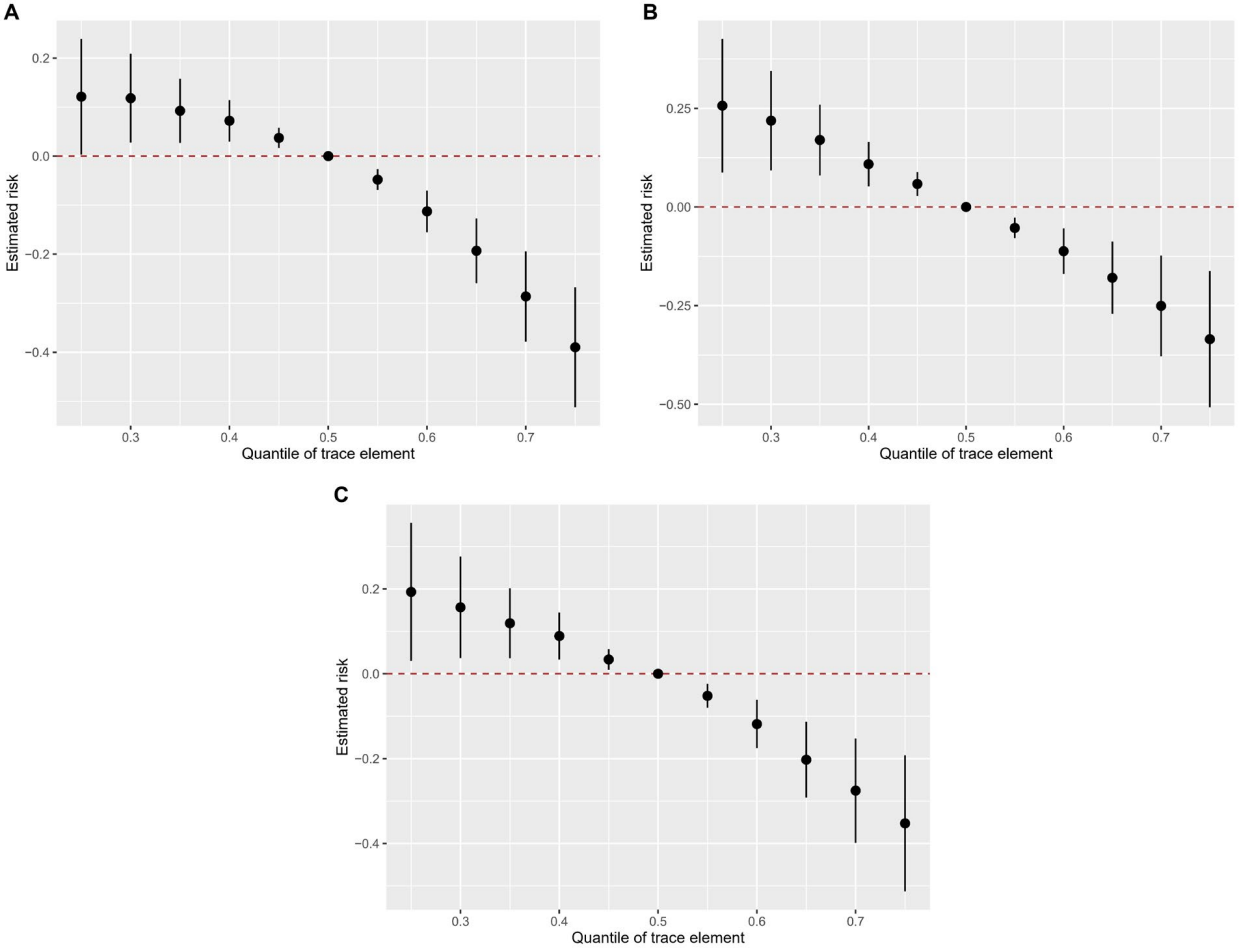
Combined effects of urinary trace elements mixture on kidney stones were estimated by BKMR models. **(A)** total population, **(B)** male subgroup, **(C)** female subgroups. Models (total) were adjusted for gender, age, race/ethnicity, education, PIR, marital status, drinking alcohol status, serum cotinine, BMI, the intake of total energy, Ca, K, Na, P, Mg, water, caffeine, and vitamin B6, C and D. This plot shows the estimated difference in kidney stones and 95%CI when all trace elements concentrations were held at particular percentiles compared to their medians.

Figure 4 shows the overall association of the trace elements mixture with the odds of kidney stones. In all participants and the two subgroups, the overall exposure-response function indicated a significant and negative association of trace elements with kidney stones.

In addition, the two-way interactions of trace elements were assessed, and 4–6 trace elements with high PIP values in each group were selected for inclusion in the two-way interactions analysis. There was a positive interaction between Ni and Pb in the whole population. The effect of Pb on kidney stones was more significant when Ni was at the higher percentile and vice versa (Supplementary Figure S4). In males, there was a negative interaction between Co and iodine. When iodine was at the higher percentile, the effect of Co on kidney stones was even less significant (Supplementary Figure S5). In females, there was an interaction between Pb and iodine. When Pb was at the higher percentile, the effect of iodine on kidney stones was more pronounced. When iodine was at the lower percentile, the association between Pb and kidney stones was positive, and as the iodine percentile increased, the association between Pb and kidney stones gradually became an “inverted U-shaped” curve (Supplementary Figure S6).

### 3.5. Sensitivity analysis

The results of sensitivity analysis are shown in Supplementary Figure S7, and the results did not substantially change after multiple imputation to all missing covariates. The OR of the trace elements was still negatively associated with kidney stones in all participants [OR = 0.57 (0.39, 0.81)]. The trace elements with higher weights are still Co, iodine, and As.

## 4. Discussion

This study analyzed the association between mixed exposure to multiple trace elements and the prevalence of kidney stones, and the interactions between elements. Combining the results of the three methods (logistic regression, qgcomp, and BKMR), we found that trace elements mixture exposure was negatively associated with the odds of kidney stones in the whole population and both male and female subgroups. Among the 13 trace elements, the elements that played the most significant role in the odds of kidney stones in the whole population were Co, iodine, and As, with a positive correlation for Co and a negative correlation for iodine and As. In males, the trace elements that played the most significant roles were As, Ni and Co, with a positive correlation for Co and negative correlations for As and Ni. In females, the trace elements that played the most important roles were Cs, iodine, and Sb, all of which were negatively correlated with the odds of kidney stones. In addition, there were interactions between iodine, Pb, and other trace elements mixtures, and two-way interactions between some trace elements.

Trace elements are ubiquitous, they exist in soil, water, air and food. The human body is constantly exposed to trace elements as a result of contact with these environmental media (26). Diseases, such as cancer and metabolic diseases may be related to excess or deficiency of trace elements (27). Excretion through the kidneys is the main way that trace elements are eliminated from the body (28). In this process, trace elements are incorporated into a urinary stone and can affect its properties (29). Trace elements also widely exist in various kidney stones. One study analyzed 31 different trace elements in 24 kidney stones using atomic absorption and found that calcium stones mainly contained iron (Fe), Pb, strontium (Sr), and zinc (Zn), and organic stones mainly contained As, Fe, and Zn (30), which means that those trace elements have an important influence on the formation of kidney stones. Zhao et al. have analyzed the associations of exposure to heavy metal mixtures with kidney stone. They got a positive association between the two (31), which was different from the result in our research. The reason for this contrast may be that their study included more toxic heavy metals like mercury, for example, while our study included potentially beneficial trace elements like iodine (which was also formalized in our study).

There have been more investigations on the link between As and Pb and kidney stones among the essential trace elements mentioned above. The toxicodynamics of As-induced kidney toxicity in experimental studies have been summarized elsewhere (32, 33). In terms of epidemiology, a previous study found a negative association between total urinary As and the risk of kidney stones in males, similar to the results of our study (17); in females, elevated As concentrations increased the risk of kidney stones (16), but our study did not find an association between the two in females, which may be related to the different number of mixed exposures in the two studies. In conclusion, there are significant differences between males and females in the correlation between kidney stones and urinary As exposure, and the specific mechanisms remain to be further elucidated.

Numerous studies on Pb have concentrated on how it affects renal function (34–36). Both modest and high levels of Pb exposure cause patients’ glomerular filtration rates to drastically decrease. Thus, the negative effects of Pb exposure on renal function are widely recognized and are supported by a large body of evidence. Regarding the association between Pb and kidney stones, there are contradictions in the existing studies. A cross-sectional survey of 1,293 Chinese participants found that high Pb exposure was positively associated with the risk of kidney stones in males and that co-exposure to Pb and Cd was also associated with the formation of kidney stones (37). In a prospective cohort study of 1,302 participants in Belgium, low-level Pb exposure was found to be associated with kidney stone formation (38). Similar findings to our analysis were reported in another investigation utilizing NHANES data: a “U-shaped” curve at higher levels of exposure and a negative correlation between Pb and kidney stone prevalence at lower levels of exposure. The reason for this phenomenon may be related to the different sensitivity to Pb in different ethnic groups, and further prospective studies are needed to figure out the relationship between Pb exposure and kidney stones in the US population.

In this study, Co was found to be strongly positively associated with kidney stone, which was similar to the previous study (31). However, analysis of the composition of trace elements in kidney stones revealed that the levels of Co in various kidney stones were very low, mostly below the detection limit (39). It indicates that Co may not be mainly involved in the composition of kidney stones, but promotes the formation of kidney stones through other pathways. It has been shown that cobalt may contribute to kidney stones by decreasing glomerular filtration rate (36) and causing acute kidney injury (40). But its specific mechanism still needs further study.

In addition, Cd was found to be significantly associated with kidney stones in previous studies, and most original studies and reviews have found a positive association between Cd exposure and kidney stones (15, 41, 42). However, the association between Cd and kidney stones occurs mostly in occupationally exposed populations (41), and dietary Cd exposure is unrelated to the prevalence of kidney stones (19). Other studies have found an “inverted U-shaped” curve relationship between the two (16, 17). However, no significant association was found between the two in this study, probably because we included more micronutrients and the different elements may interact with each other.

The results of this study also showed that iodine, Ni, and Cs all have important effects on kidney stones. However, there are few studies on the effects of these trace elements on kidney stones. Urinary Ni excretion was significantly lower in patients with active kidney stones compared to patients with previous kidney stones and healthy individuals who had never had kidney stones (43). Some Ni particles were also found in kidney stones (44), and Ni may inhibit the crystallization process of calcium oxalate (45, 46). All these results indicate the importance of Ni in the pathogenesis of new kidney stone formation. A study using NHANES 2011–2016 data found an interaction between Cs and manganese (Mn) in terms of their effects on kidney function (47). Few studies have examined the effects of iodine and Cs on kidney stones, but in our study, we found a negative association between iodine exposure and the odds of kidney stones in the whole population, and in both male and female subgroups; Cs was also negatively associated with the prevalence of kidney stones in females. We believe these findings warrant further prospective cohort studies.

Additionally, this study investigated the interactions of trace elements on the odds of kidney stones. Interactions were found between iodine and Pb and other trace elements mixture, and two-way interactions were found between many trace elements, such as Ni and Pb (whole population), Co and iodine (male), and Pb and iodine (female). However, we judged the interactions only by graphs and lack of interaction parameters, which can lead to subjective misjudgment. Other more suitable methods could be chosen to further investigate the interactions of trace elements on kidney stones specifically.

In subgroup analysis, different elements affecting differences between females and males. The reason might be the compositions of kidney stones are different between males and females. Compared to men, women have a lower proportion of CaOx stones and uric acid (UA) stones and a higher proportion of hydroxyapatite (HA) in kidney stones (48, 49). The amount of trace elements contained in different types of kidney stones is also different and it was statistically significant (39). Besides, the difference might be mediated through sex hormones. Different trace elements had different effects on the production of sex hormones in men or women (50), and changes in sex hormones could further promote or inhibit the development of kidney stones (51). The root reason needs further mechanistic researches.

Our study has some strengths. Firstly, 3 methods, logistic regression, qgcomp, and BKMR, were used to analyze the effect of trace elements on kidney stones. Secondly, all urinary trace element concentrations available in the NHANES database with a detection rate > 70% were included in the analysis, which is similar to real human exposure. Thirdly, the impact of each trace element on kidney stones was in the same direction by the three methods and the results were similar through the three different methods, especially in the elements with higher contributions to kidney stone. Fourthly, the three methods produced generally consistent results, demonstrating the robustness of the findings.

Inevitably, this study also has some limitations: firstly, the data in the NHANES database are cross-sectional, so it is difficult to infer a causal relationship between trace elements exposure and the odds of kidney stones. Secondly, since the trace elements we intended to study include Ni, only data from NHANES 2017–2018 were included. Thirdly, the concentrations of trace elements in urine may not reflect the actual exposure of the human body. Fourthly, self-reported kidney stones diagnosis may potentially affect the credibility of the results. Fifthly, BKMR and qgcomp cannot consider the sample weights for complex sampling, which may lead to inaccurate results. Sixthly, some unknown or residual mixes cannot be fully adjusted due to information acquisition limitations.

## 5. Conclusion

We found a negative association between trace elements mixture exposure and the prevalence of kidney stones. We also identified elements such as Co, As, iodine, Ni, and Cs that were significantly associated with the odds of kidney stones and found that there was a positive interaction between Ni and Pb in the whole population. Given the limitations of this study and the fact that the research was an exploratory study, these results need to be interpreted with caution and further investigations are needed to support our findings.

## Data availability statement

Publicly available datasets were analyzed in this study. This data can be found here: [NHANES] repository (https://www.cdc.gov/nchs/nhanes/).

## Ethics statement

The studies involving humans were approved by the studies involving human participants were reviewed and approved by the [National Center for Health Statistics (NCHS) Ethics Review Board, Centers for Disease Control and Prevention, 1,600 Clifton Road Atlanta, GA 30329–4,027 USA]. The studies were conducted in accordance with the local legislation and institutional requirements. The participants provided their written informed consent to participate in this study.

## Author contributions

XW: Conceptualization, Methodology, Software, Visualization, Writing – original draft, Data curation. JZ: Software, Validation, Writing – review & editing. ZM: Writing – original draft. YY: Writing – original draft. YD: Writing – original draft. SC: Writing – original draft. XS: Writing – original draft. CO: Writing – original draft. JP: Writing – original draft. XH: Supervision.
