# Supplementary material for: Association and interactions between mixed exposure to trace elements and the prevalence of kidney stones: a study of NHANES 2017–2018
Source: Front Public Health. 2023 Oct 26;11:1251637. doi: 10.3389/fpubh.2023.1251637 (PMC10642183; doi:10.3389/fpubh.2023.1251637)
Supplement: Supplementary file 1 [file Data_Sheet_1.docx]

Supplementary Material

Association and interaction between mixed exposure to trace elements and the prevalence of kidney stones: A study of NHANES 2017-2018

**Xiao Wang^1^, Jia Zhang^1^, Zhibin Ma^1^, Yaya Yang^1^, Ying Dang^1^, Shuting Cao^1^, Xiaoru Shi^1^, Changping Ouyang^1^, Jinhua Pan^1^, Xiaobin Hu^1^***

1. Institute of Epidemiology and Health Statistics, School of Public Health, Lanzhou University, Lanzhou, China

*** Correspondence:** Xiaobin Hu: [lzhuxb@126.com](mailto:lzhuxb@126.com)

Address for correspondence

Xiaobin Hu, Prof.

Institute of Epidemiology and Health Statistics, School of Public Health, Lanzhou University

No.199, Donggang West Road, Chengguan District, Lanzhou, Gansu Province, 730000, China

- **Table S1** STROBE Statement—Checklist of items that should be included in reports of ***cross-sectional studies.***

|  | Item No | Recommendation | Page No. |
| --- | --- | --- | --- |
| **Title and abstract** | 1 | (*a*) Indicate the study’s design with a commonly used term in the title or the abstract | Page 1, 2 |
|  |  | (*b*) Provide in the abstract an informative and balanced summary of what was done and what was found | Page 2 |
| Introduction | | |  |
| Background/rationale | 2 | Explain the scientific background and rationale for the investigation being reported | Page 3 |
| Objectives | 3 | State specific objectives, including any prespecified hypotheses | Page 4 |
| Methods | | |  |
| Study design | 4 | Present key elements of study design early in the paper | Page 5 |
| Setting | 5 | Describe the setting, locations, and relevant dates, including periods of recruitment, exposure, follow-up, and data collection | Page 5, 7 |
| Participants | 6 | (*a*) Give the eligibility criteria, and the sources and methods of selection of participants | Page 5  Figure 1 |
| Variables | 7 | Clearly define all outcomes, exposures, predictors, potential confounders, and effect modifiers. Give diagnostic criteria, if applicable | Page 7 |
| Data sources/ measurement | 8* | For each variable of interest, give sources of data and details of methods of assessment (measurement). Describe comparability of assessment methods if there is more than one group | Page 5 |
| Bias | 9 | Describe any efforts to address potential sources of bias | Page 7 |
| Study size | 10 | Explain how the study size was arrived at | Page 5 |
| Quantitative variables | 11 | Explain how quantitative variables were handled in the analyses. If applicable, describe which groupings were chosen and why | Page 8 |
| Statistical methods | 12 | (*a*) Describe all statistical methods, including those used to control for confounding | Page 8,9 |
|  |  | (*b*) Describe any methods used to examine subgroups and interactions | Page 8, 9 |
|  |  | (*c*) Explain how missing data were addressed | Page 9 |
|  |  | (*d*) If applicable, describe analytical methods taking account of sampling strategy |  |
|  |  | (*e*) Describe any sensitivity analyses | Page 9 |
| Results | | |  |
| Participants | 13* | (a) Report numbers of individuals at each stage of study—eg numbers potentially eligible, examined for eligibility, confirmed eligible, included in the study, completing follow-up, and analysed | Page 10 |
|  |  | (b) Give reasons for non-participation at each stage | Figure 1 |
|  |  | (c) Consider use of a flow diagram | Figure 1 |
| Descriptive data | 14* | (a) Give characteristics of study participants (eg demographic, clinical, social) and information on exposures and potential confounders | Page 10  Table 1 |
|  |  | (b) Indicate number of participants with missing data for each variable of interest | Figure 1 |
| Outcome data | 15* | Report numbers of outcome events or summary measures | Page 10 |
| Main results | 16 | (*a*) Give unadjusted estimates and, if applicable, confounder-adjusted estimates and their precision (eg, 95% confidence interval). Make clear which confounders were adjusted for and why they were included | Page 13-22  Table 2, 4  Figure 2-4 |
|  |  | (*b*) Report category boundaries when continuous variables were categorized | Table S3 |
|  |  | (*c*) If relevant, consider translating estimates of relative risk into absolute risk for a meaningful time period | - |
| Other analyses | 17 | Report other analyses done—eg analyses of subgroups and interactions, and sensitivity analyses | Page 13, 22  Table 3  Figure S7 |
| Discussion | | |  |
| Key results | 18 | Summarise key results with reference to study objectives | Page 23-25 |
| Limitations | 19 | Discuss limitations of the study, taking into account sources of potential bias or imprecision. Discuss both direction and magnitude of any potential bias | Page 26 |
| Interpretation | 20 | Give a cautious overall interpretation of results considering objectives, limitations, multiplicity of analyses, results from similar studies, and other relevant evidence | Page 23-26 |
| Generalisability | 21 | Discuss the generalisability (external validity) of the study results | Page 27 |
| Other information | | |  |
| Funding | 22 | Give the source of funding and the role of the funders for the present study and, if applicable, for the original study on which the present article is based | Page 28 |

*Give information separately for exposed and unexposed groups.

- **Table S2** Distributions of urine trace elements in the study population.

| Trace element (ug/L) | Lower detection limit | Detection rate (%) | Median | Interquartile range |
| --- | --- | --- | --- | --- |
| Ni | 0.31 | 92.68 | 1.15 | 1.24 |
| Mo | 0.80 | 100.00 | 33.69 | 41.98 |
| Tl | 0.02 | 99.76 | 0.16 | 0.16 |
| Pb | 0.03 | 99.84 | 0.32 | 0.39 |
| Ba | 0.06 | 99.60 | 0.92 | 1.54 |
| Co | 0.02 | 99.84 | 0.40 | 0.40 |
| Cs | 0.09 | 100.00 | 4.44 | 3.87 |
| Sb | 0.02 | 78.14 | 0.04 | 0.05 |
| Sn | 0.09 | 92.85 | 0.48 | 0.87 |
| Tu | 0.02 | 85.45 | 0.06 | 0.08 |
| As | 0.23 | 100.00 | 6.24 | 10.23 |
| I | 2.40 | 100.00 | 116.80 | 139.30 |
| Cd | 0.04 | 95.74 | 0.22 | 0.31 |

- **Table S3** The category boundaries of all 13 trace elements in this study.

| Trace  element | Q1  (ug/g cr) | Q2  (ug/g cr) | Q3  (ug/g cr) | Q4  (ug/g cr) |
| --- | --- | --- | --- | --- |
| Ni | < 0.74 | 0.74 - 1.13 | 1.13 - 1.75 | ≥ 1.75 |
| Mo | < 21.94 | 21.94 - 34.11 | 34.11 - 51.40 | ≥ 51.40 |
| Tl | < 0.11 | 0.11 - 0.16 | 0.16 - 0.22 | ≥ 0.22 |
| Pb | < 0.20 | 0.20 - 0.33 | 0.33 - 0.53 | ≥ 0.53 |
| Ba | < 0.50 | 0.50 - 0.99 | 0.99 - 1.90 | ≥ 1.90 |
| Co | < 0.26 | 0.26 - 0.39 | 0.39 - 0.58 | ≥ 0.58 |
| Cs | < 3.10 | 3.10 - 4.24 | 4.24 - 5.90 | ≥ 5.90 |
| Sb | < 0.03 | 0.03 - 0.04 | 0.04 - 0.07 | ≥ 0.07 |
| Sn | < 0.26 | 0.26 - 0.49 | 0.49 - 0.96 | ≥ 0.96 |
| Tu | < 0.03 | 0.03 - 0.06 | 0.06 - 0.09 | ≥ 0.09 |
| As | < 3.39 | 3.39 - 5.94 | 5.94 - 13.58 | ≥ 13.58 |
| I | < 69.80 | 69.80 - 114.60 | 114.60 - 212.05 | ≥ 212.05 |
| Cd | < 0.12 | 0.12 - 0.22 | 0.22 - 0.42 | ≥ 0.42 |


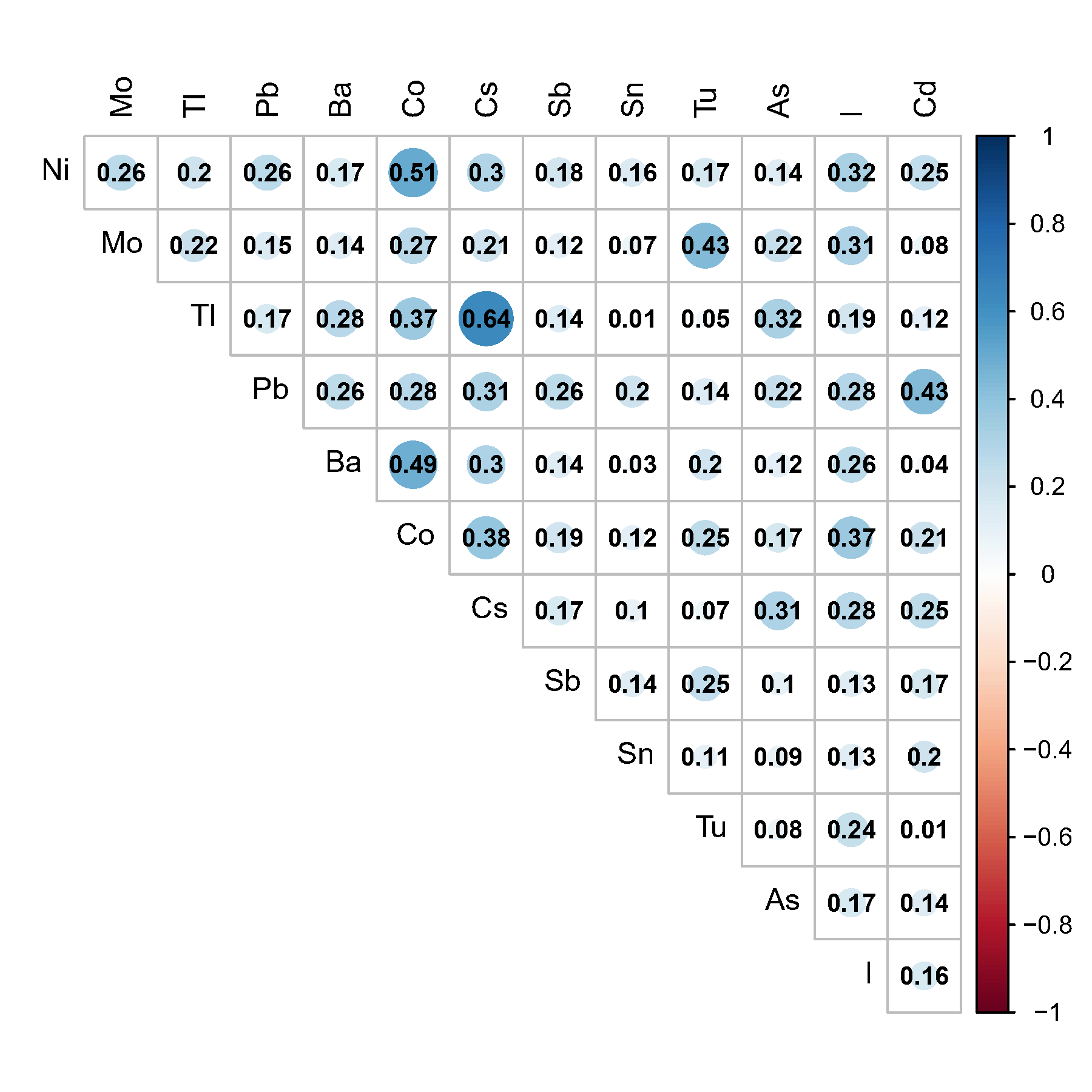


- Figure S1 Spearmen’s correlation matrix among urinary trace elements in the study population.


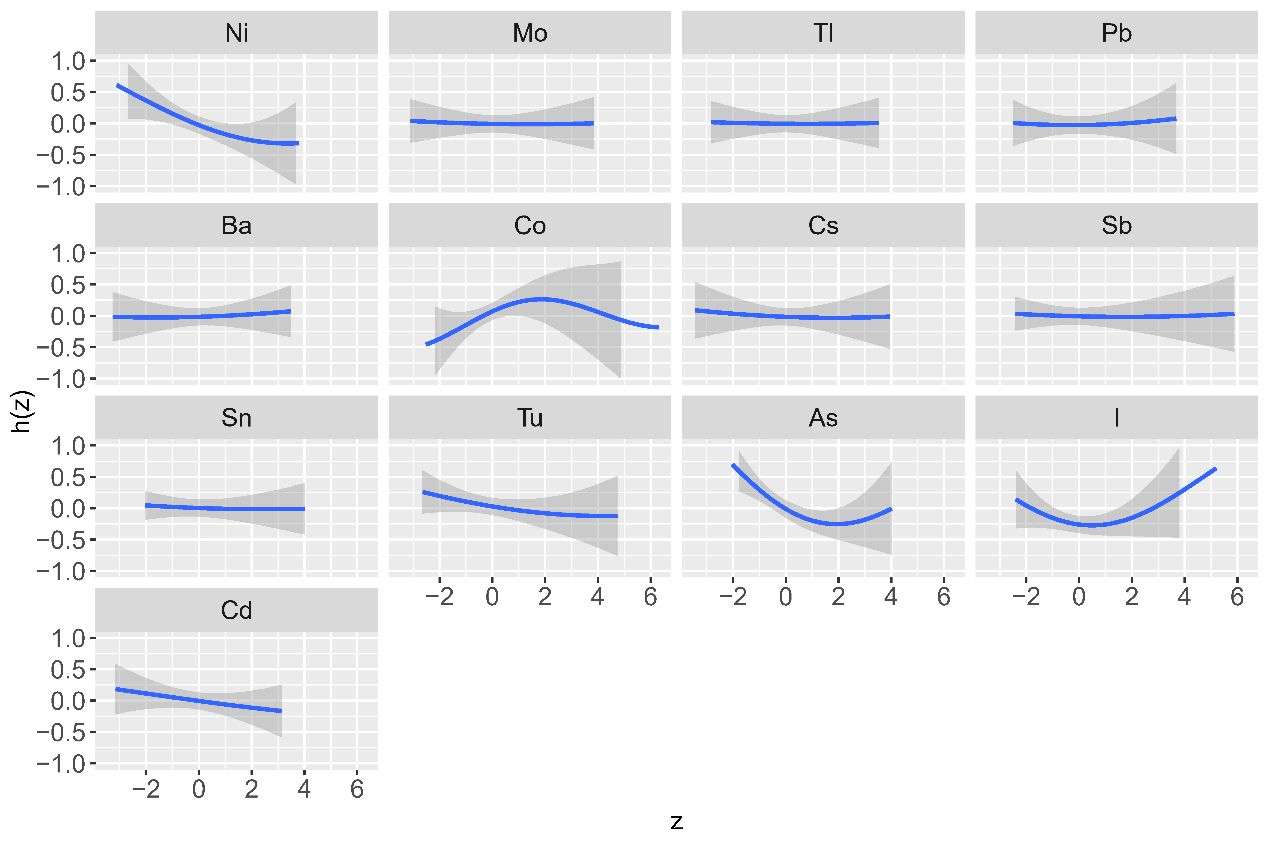


- **Figure S2 Univariate exposure-response functions for each trace element with other elements fixed at the median (male).** The results were assessed by the Bayesian Kernel Machine Regression (BKMR) models. Models were adjusted for age, race/ethnicity, education, PIR, marital status, drinking alcohol status, serum cotinine, BMI, the intake of total energy, Ca, K, Na, P, Mg, water, caffeine and vitamin B6, C and D.


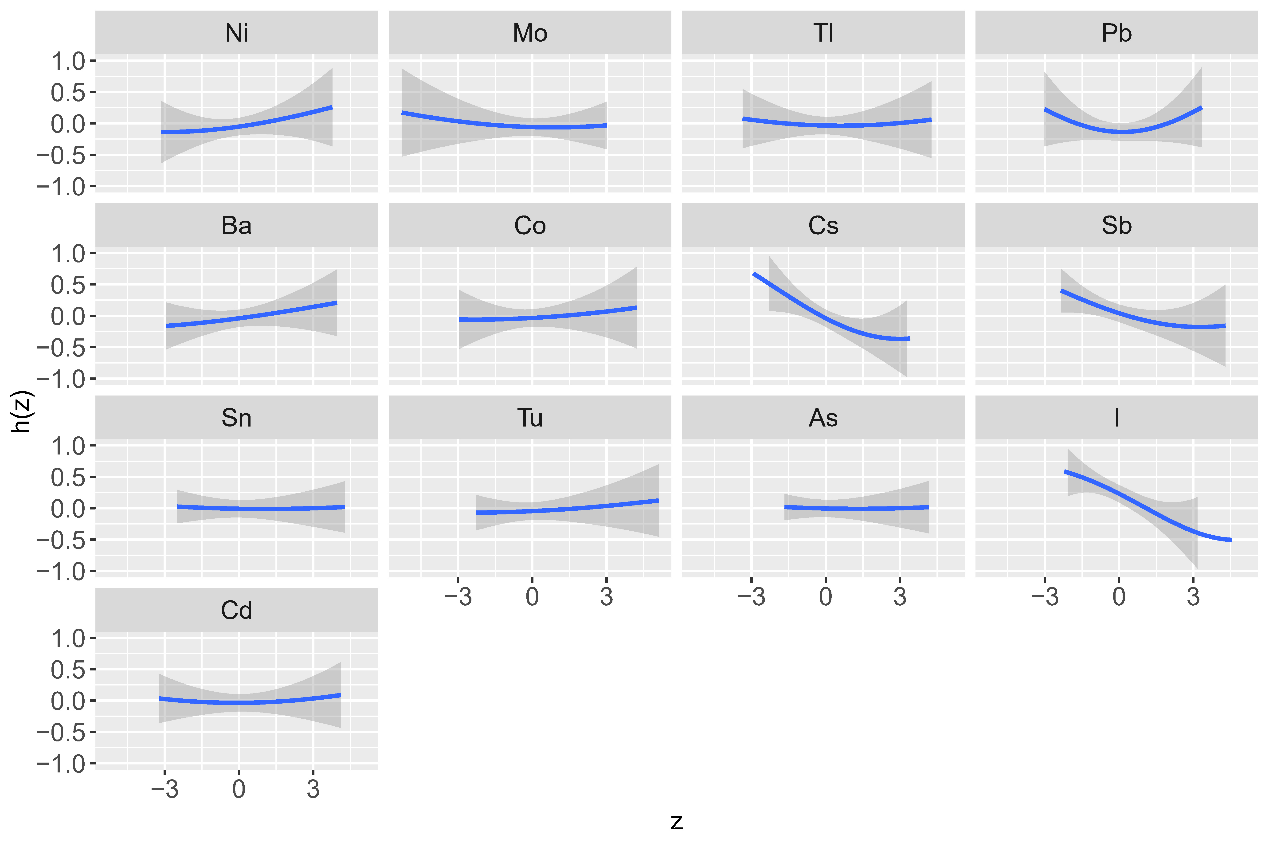


- **Figure S3 Univariate exposure-response functions for each trace element with other elements fixed at the median (female).** The results were assessed by the Bayesian Kernel Machine Regression (BKMR) models. Models were adjusted for age, race/ethnicity, education, PIR, marital status, drinking alcohol status, serum cotinine, BMI, the intake of total energy, Ca, K, Na, P, Mg, water, caffeine and vitamin B6, C and D.


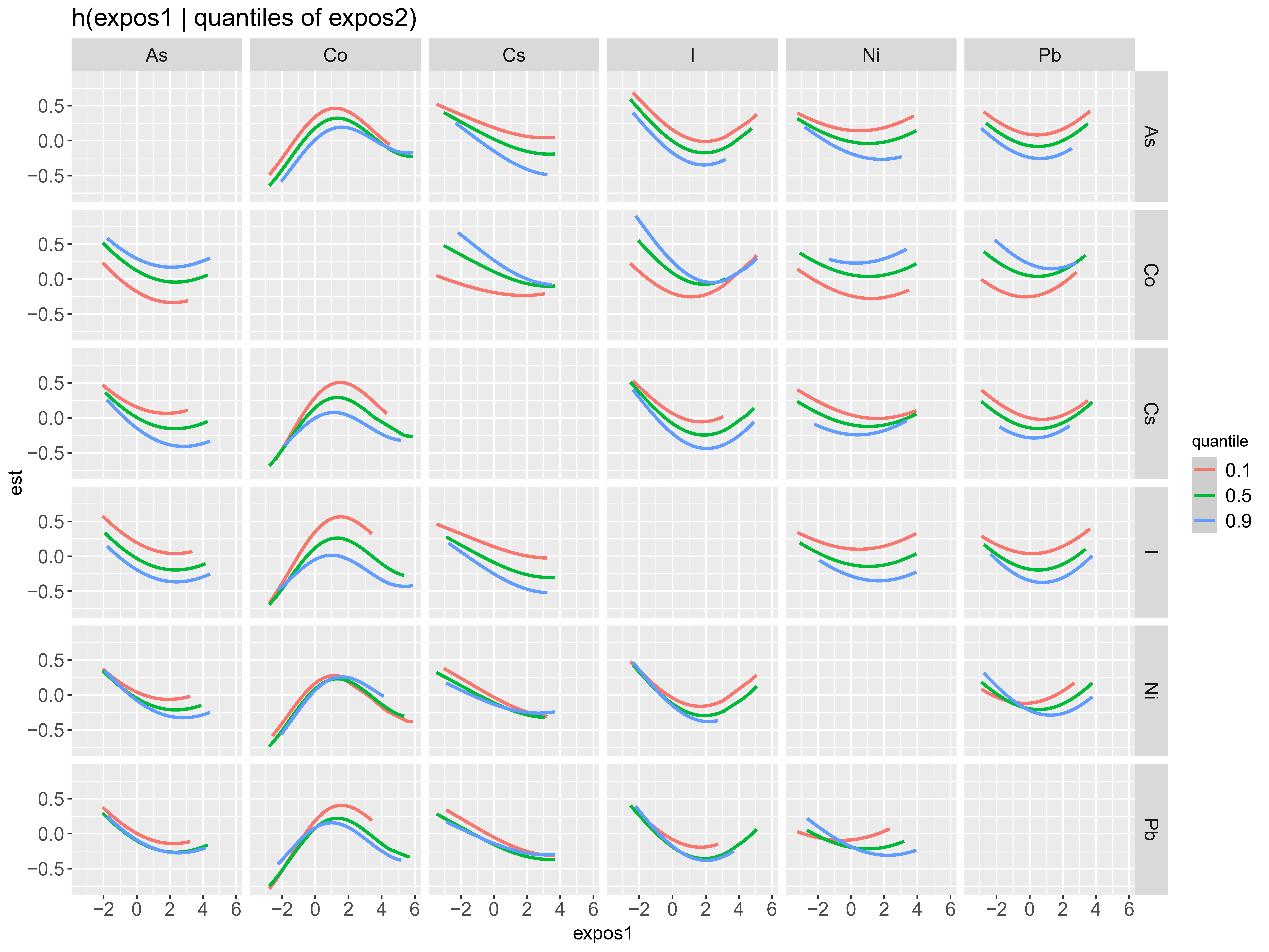


- **Figure S4 The interaction function among two trace elements with other elements fixed at the median (total).** The results were assessed by the Bayesian Kernel Machine Regression (BKMR) models. Models were adjusted for sex, age, race/ethnicity, education, PIR, marital status, drinking alcohol status, serum cotinine, BMI, the intake of total energy, Ca, K, Na, P, Mg, water, caffeine and vitamin B6, C and D.


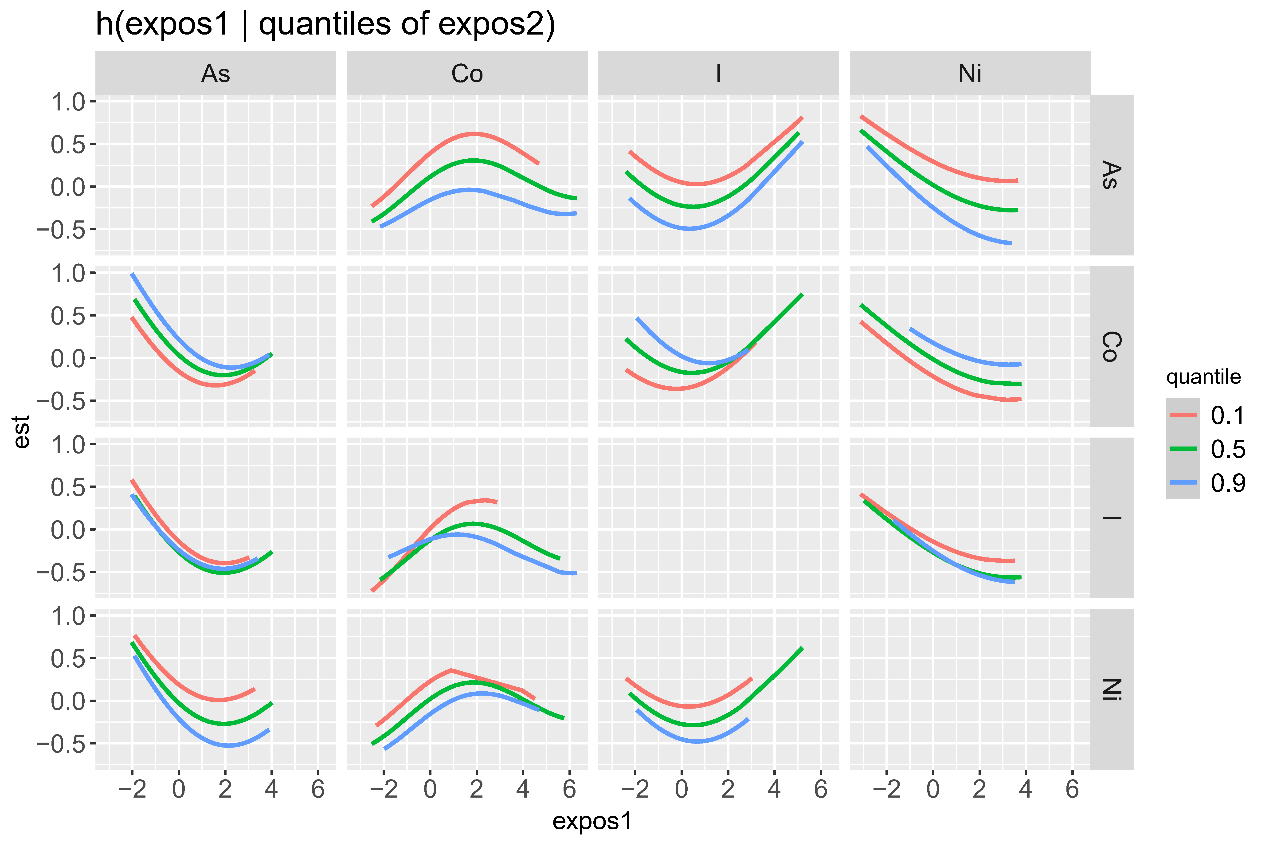


- **Figure S5** **The interaction function among two trace elements with other elements fixed at the median (male).** The results were assessed by the Bayesian Kernel Machine Regression (BKMR) models. Models were adjusted for age, race/ethnicity, education, PIR, marital status, drinking alcohol status, serum cotinine, BMI, the intake of total energy, Ca, K, Na, P, Mg, water, caffeine and vitamin B6, C and D.


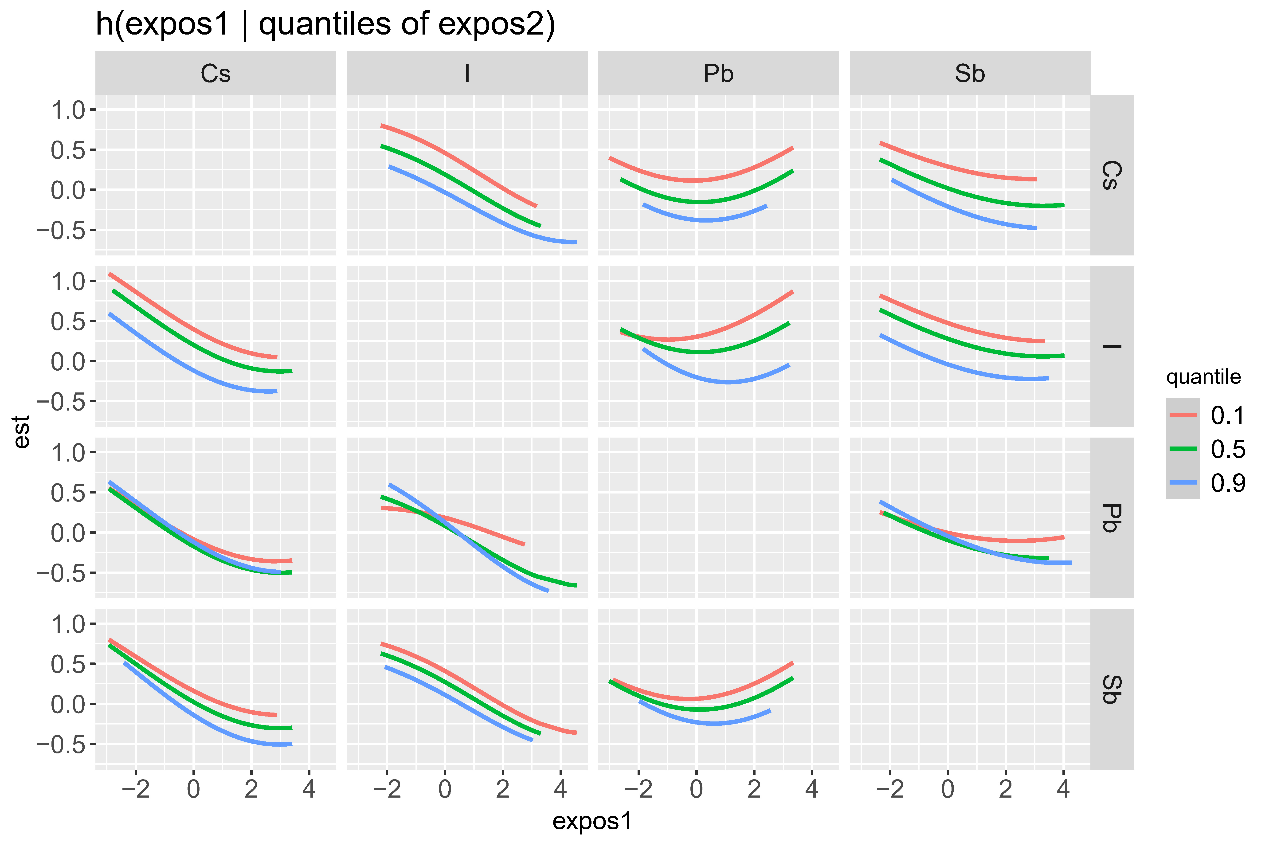


- **Figure S6 The interaction function among two trace elements with other elements fixed at the median (female).** The results were assessed by the Bayesian Kernel Machine Regression (BKMR) models. Models were adjusted for age, race/ethnicity, education, PIR, marital status, drinking alcohol status, serum cotinine, BMI, the intake of total energy, Ca, K, Na, P, Mg, water, caffeine and vitamin B6, C and D.


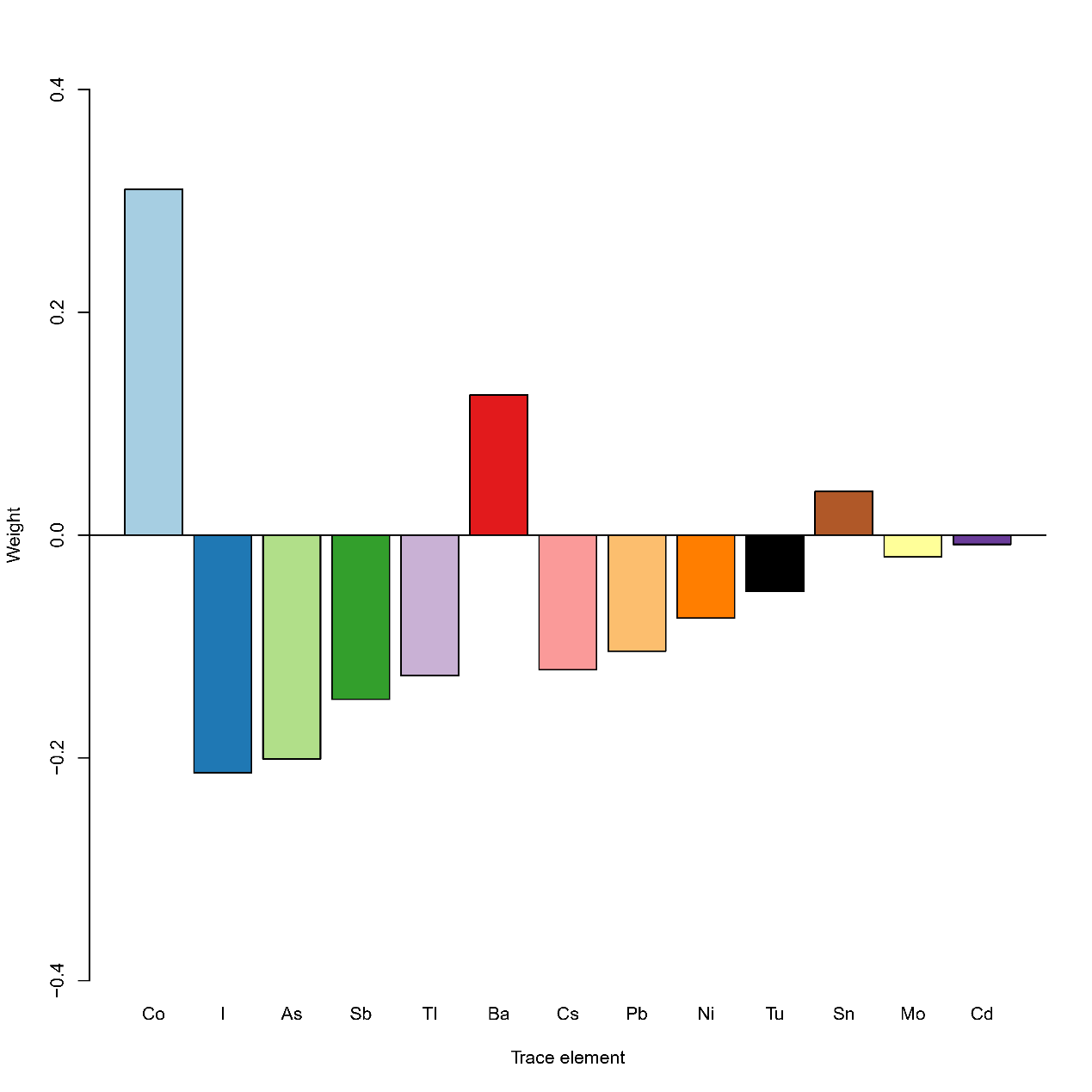


- **Figure S7 Estimated weighted values of trace elements for kidney stone by qgcomp models after multiple imputation to all missing covariates.** Model was adjusted for age, race/ethnicity, education, PIR, marital status, drinking alcohol status, serum cotinine, BMI, the intake of total energy, Ca, K, Na, P, Mg, water, caffeine and vitamin B6, C and D.
